# Pain Clinic in Tibet, China: A Single-Center Retrospective Study

**DOI:** 10.1155/2019/9161906

**Published:** 2019-01-14

**Authors:** Le Shen, Xin Zhang, Dawawuzhu, Labaciren, Yuelun Zhang, Zhonghuang Xu, Yuguang Huang

**Affiliations:** ^1^Department of Anesthesiology, Peking Union Medical College Hospital, Chinese Academy of Medical Sciences and Peking Union Medical College, Beijing, China; ^2^Department of Anesthesiology, Tibet Autonomous Region People's Hospital, Lhasa, China; ^3^Central Research Laboratory, Peking Union Medical College Hospital, Chinese Academy of Medical Sciences and Peking Union Medical College, Beijing, China

## Abstract

Pain disease is a worldwide problem.The prevalence of chronic pain in developed and developing countries has been reported in some published research. However, little knowledge of situation of pain clinic in Tibet is known. Tibet Autonomous Region People's Hospital established the first pain clinic in Tibet. This study collected and analyzed the data of medical records of pain clinic in Tibet Autonomous Region People's Hospital from September 2017 to August 2018. The results showed that the total amounts of patients visiting pain clinic were very small, the most common pain diseases were postherpetic neuralgia and sciatica, and more female patients visited the pain clinic than male patients. All these results indicate that the hospital and government need to pay more attention to the development and promotion of pain medicine in Tibet to make Tibetans being accessed to high-quality pain clinic service.

## 1. Introduction

Chronic pain is a worldwide problem, which creates huge burden on patients and society. The prevalence of chronic pain in Europe, America, and Australia is 12%–30%, 11%, and 17.1%–20%, respectively [1–3]. In China, some similar studies had been performed in recent years. Zhang et al. did a cross-sectional study between the prevalence of chronic pain and academic pressure in adolescents in Shanghai, and the results showed that the prevalence of headache, abdominal pain, neck and shoulder pain, and low back pain was 30.3%, 20.9%, 32.8%, and 41.1%, respectively, which indicated that the high-school students were experiencing huge academic pressure [4]. Jackson et al. designed a study to assess the prevalence of chronic pain among adults in Chongqing. Their investigation found that 25.8% interviewees suffered from chronic pain [5]. In the Chaoyang district of Beijing, the prevalence of chronic pain was 52.99% [6], much higher than the data mentioned above. However, little is known about the epidemiology of pain diseases in some special area, for example, Qinghai-Tibet plateau of China.

The Qinghai-Tibet plateau of China is a plateau region with an average elevation of 3,000 meters above the sea level, and it is the highest region on earth. For Tibet Autonomous Region (TAR), China, it occupies 1.23 million square kilometers with a population of about 3.3 million. According to the worldwide data, there might be at least 350,000 chronic pain patients in TAR. However, altogether about 150 anesthesia practitioners, including anesthesiologist and nurse anesthetists, are providing anesthesia and pain medical services in Tibet Autonomous Region now. So, the diagnosis and management of chronic had been underestimated or ignored for years.

On September 28, 2017, the first pain clinic of Tibet Autonomous Region was set up in Tibet Autonomous Region People's Hospital (TARPH), Lhasa. TARPH is one of the biggest class A tertiary general hospitals in Tibet Autonomous Region and committed to the medical services, medical education/teaching, research, and prevention of the whole area of Tibet. Therefore, we conducted this study to understand the situation of pain clinic in TAR, China, in three aspects: first, the pain disease categorization of outpatients; second, the risk factors for pain; and third, comparing the situation of pain diagnosis and treatment with other areas of China.

## 2. Materials and Methods

This investigation was a retrospective hospital-based, case series study approved by the TARPH Institutional Review Board. All the chronic pain patients of TARPH pain clinic from September 2017 to August 2018 were enrolled retrospectively. The outpatient records including demographic data, clinical manifestation, diagnosis, and treatment were collected.

For statistical analysis, continuous data were expressed as mean (±standard deviation). Histogram was used to check the normality of data. Statistical analysis was performed using SPSS 19.0.

## 3. Results

### 3.1. Basic Information of the Pain Clinic Records

Totally, 37 medical records of pain clinic of TARPH from September 2017 to August 2018 were collected. Among them, 29 patients visited the pain clinic only once, 6 patients for two times, and 2 patients for three times. The average age of these 37 patients was 54.8 years, with the male to female sex ratio of 16/21.

### 3.2. Pain Disease Categorization and Demographic Characteristics of the Pain Clinic Records

We divided the patients into four groups according to the locations of pain. Some patients suffered from pain of multiple sites. (Table 1). Pain of trunk and lower extremity consisted of majority of the cases. Except the abdominal pain, low back pain, and lower extremity pain, more females suffered from pain diseases than males.

**Figure 1:**
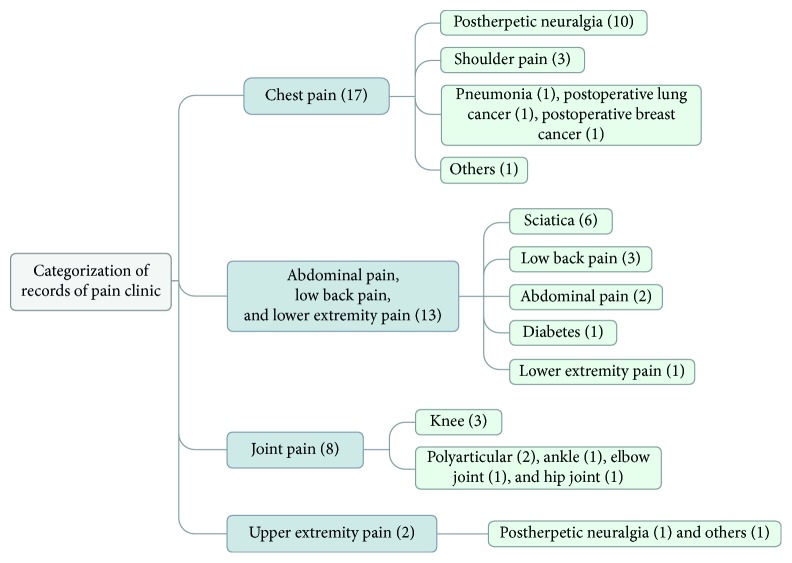
Categorization of medical records of pain clinic.

After analyzing all the cases of the medical records, we found that postherpetic neuralgia and sciatica consisted of nearly half of the cases. However, the etiological factors of some patients were still not clear. The detail categorization of these records is shown in Figure 1.

### 3.3. Patients Diagnosed with Postherpetic Neuralgia

Totally, 11 (29.7%) patients were diagnosed with postherpetic neuralgia in TARPH pain clinic. The average age of these 11 patients was 70.4 years, with the male to female sex ratio of 5/6. Almost all the patients came to see pain physicians more than one month after the onset of herpetic zoster. Only one patient received treatment of gabapentinoids from dermatologists. Six out of eleven postherpetic neuralgia patients visited the pain clinic for more than one time (Table 2).

## 4. Discussion

Medical aid for Tibet Autonomous Region is an important strategic decision of the country. The government gives top priority to the improvement of Tibetan's livelihoods. The medical experts coming to Tibet not only saved the life of Tibetans but also contributed to the unity and stability of the country. As anesthesiologists, we should try the best to support this strategy in our subject areas. Despite the aid of clinical anesthesia for Tibet, we should also pay attention to pain medicine in Tibet. The pain clinic of Tibet Autonomous Region People's Hospital is the first pain clinic in Tibet area. This is the first study in China to investigate the situation of pain clinic in the Qinghai-Tibet plateau. Tibet Autonomous Region People's Hospital is a class A tertiary comprehensive hospital and established the first pain clinic in Tibet; therefore, these data could represent the situation of pain clinic in Tibet area. Comparing with the pain clinics of other provincial capital hospitals in China, the outpatient amounts of Tibet Autonomous Region People's Hospital were so small. Some reasons may explain this phenomenon. Firstly, the inhabitants of Tibet may not be aware of the existence of pain clinic, so they may choose other departments as their first consultation departments. Secondly, the pain medicine is not the focus of the development of this hospital, and it was short of professionally trained pain physicians. Thirdly, the hospital may lack fund to introduce some necessary equipment to carry out pain diagnosis and treatment.

As all the cases were outpatients, we cannot estimate the prevalence of chronic pain in Tibet. The data of these medical records showed that the mean age of these patients was 54.81 ± 15.66, older than the data (39.5 ± 15.67) presented by Jackson et al. [5]. It may indicate that the inhabitants of Tibet have tolerated pain diseases for a long time before they visited the pain clinic for the first time. It may delay treatment and cause more consumption of medical resources. However, this phenomenon may also indicate that the Tibetans are more physically healthy. Further study needs to be done to confirm the reason why the mean age of pain clinic patients in Tibet was older than the data presented by Jackson et al.

From the data of this study, we could find that more females visit the pain clinic than males. This phenomenon coincides with some published articles [5–8]. The underlying mechanism is not clear. Some animal research indicated that ovarian hormones may contribute to the pain development [9, 10].

We found that postherpetic neuralgia and sciatica are the most common diagnosis of pain clinic. These two kinds of diseases could significantly influence the daily life of patients and make the patients to seek for medical help. The frequency and severity of PHN increase with advancing age, occurring in 20% of people aged 60–65 years who have had acute HZ, and in more than 30% of people aged >80 years [11]. The effect of female gender seemed protective in studies in which the mean age was ≥60 years, compared with among studies with mean age <60 years, for which female gender increased the risk of PHN [12]. Our results also show that most PHN patients in Tibet are older than 70 years (7/11), but no gender association was found. The first-line analgesic medication for PHN is gabapentinoids [13]; however, patients diagnosed with postherpetic neuralgia had not been effectively treated before they visited the pain clinic. Therefore, the pain physicians should pay more attention to the update of diagnosis and treatments of these pain conditions, and on the other hand, local physicians should receive more training in pain medicine. Local hospital should supply the basic drugs and equipment to ensure whether the clinical practice is going well.

Small sample size is the major limitation of this study. However, it suggested that the hospital and the government should establish some policy to develop and promote pain medicine in Tibet and let more Tibet inhabitants know that they can seek help from pain clinic if they are suffering from pain symptoms.

Another limitation is that the data that we could use are age, sex, and diagnoses and we could not perform deep analysis of the data. This fact also reflects the urgency of promotion of pain medicine, supplying the relative drugs, equipment, and tests in this hospital to enrich the data of medical records. If the hospital has a pain ward, we could also collect much more data than before.

## 5. Conclusions

This study revealed the situation of pain medicine in Tibet. Both the government and the hospital should make efforts to develop pain medicine to make Tibetans being accessed to high-quality pain clinic service.

## Figures and Tables

**Table 1:** Subgroup analysis of different pain diseases.

Pain diseases	Number	Age (mean ± SD)	Sex (male/female)
Pain of anterior and posterior aspects of the thorax	17	64.06 ± 15.46	7/10
Abdominal pain, low back pain, and lower extremity pain	13	47.08 ± 11.64	8/5
Joint pain	8	44.63 ± 9.04	2/4
Upper extremity pain	2	53 ± 4.24	1/1

**Table 2:** Summary of postherpetic neuralgia patients.

No.	Gender	Age	Spinal cord level	Duration from onset to pain clinic	Previous treatment	Follow-up
1	M	56	C8-T1	9 m	Vitamin B6, Vitamin B12	1
2	F	82	T levels	Not known	No	No
3	F	75	T6-T8	4 m	No	1
4	F	77	T8	40 m	No	1
5	F	62	T11-T12	1.5 m	Antiviral gabapentin	2
6	F	71	T levels	Not known	COX-2	No
7	M	75	T levels	Not known	COX-2	No
8	M	62	T10-T12	3 m	No	1
9	F	82	T levels	Not known	No	No
10	M	63	T8-T9	1 m	Antiviral	1
11	M	70	T10	1.5 m	Antiviral	No

## Data Availability

The datasets analyzed during the current study are available from the corresponding author on reasonable request.
